# Evidence for Gene Flow between Two Sympatric Mealybug Species (Insecta; Coccoidea; Pseudococcidae)

**DOI:** 10.1371/journal.pone.0088433

**Published:** 2014-02-11

**Authors:** Hofit Kol-Maimon, Murad Ghanim, José Carlos Franco, Zvi Mendel

**Affiliations:** 1 Department of Entomology, Volcani Center (ARO), Bet Dagan, Israel; 2 Departamento de Ciências e Engenharia de Biossistemas/Centro de Estudos Florestais, Instituto Superior de Agronomia, Universidade de Lisboa, Lisboa, Portugal; University of Massachusetts, United States of America

## Abstract

Occurrence of inter-species hybrids in natural populations might be evidence of gene flow between species. In the present study we found evidence of gene flow between two sympatric, genetically related scale insect species – the citrus mealybug *Planococcus citri* (Risso) and the vine mealybug *Planococcus ficus* (Signoret). These species can be distinguished by morphological, behavioral, and molecular traits. We employed the sex pheromones of the two respective species to study their different patterns of male attraction. We also used nuclear ITS2 (internal transcribed spacer 2) and mitochondrial COI (Cytochrome c oxidase sub unit 1) DNA sequences to characterize populations of the two species, in order to demonstrate the outcome of a possible gene flow between feral populations of the two species. Our results showed attraction to *P. ficus* pheromones of all tested populations of *P. citri* males but not vice versa. Furthermore, ITS2 sequences revealed the presence of ‘hybrid females’ among *P. citri* populations but not among those of *P. ficus*. ‘hybrid females’ from *P. citri* populations identified as *P. citri* females according to COI sequences. We offer two hypotheses for these results. 1) The occurrence of phenotypic and genotypic traits of *P. ficus* in *P. citri* populations may be attributed to both ancient and contemporary gene flow between their populations; and 2) we cannot rule out that an ancient sympatric speciation by which *P. ficus* emerged from *P. citri* might have led to the present situation of shared traits between these species. In light of these findings we also discuss the origin of the studied species and the importance of the pherotype phenomenon as a tool with which to study genetic relationships between congener scale insects.

## Introduction

Genetic isolation between species in areas of sympatry may occur via three types of sex barriers [1]. The first and most common type is ‘ecological isolation’, which includes adaptation to different habitats and different seasonal phenotypic features, leading to isolation between potential mates in space and time. There is evidence that ecological isolations can be breached under laboratory conditions following interbreeding [2], [3], [4]. The second type - ‘postzygotic isolation’ is created by copulation and fertilization with an outcome of non-fertile offspring [5]. The third type - ‘behavioral isolation’ refers to cases where potential mates meet but do not copulate because courtship behavior patterns do not mesh [6], [7], [8].

Hybrid speciation is one form of sympatric speciation, defined as the occurrence of new species as an outcome of interbreeding between two or more species [9]. Hybrid speciation begins with the creation of a hybrid zone, in which genetically distinct groups meet and produce individuals of mixed ancestry [10], [11], [12]. Hybrid individuals are restricted to the hybrid zone as long as they display low fitness and survival incapability [11]. When the habitat or the population gene pool changes, hybrid zone can be breached and hybrid speciation may begin [11], [9].

Two mealybug species, the citrus mealybug *Planococcus citri* (Risso) and the vine mealybug *Planococcus ficus* (Signoret) are key pests of a wide range of agricultural crops, and they share many host plants and habitats. Both mealybug species are known to transmit plant viruses and to secrete honeydew on which sooty mold develops, thereby causing severe economic losses worldwide [23], [24], [25], [26]. The taxonomic status of these species has been disputed for almost two centuries. In 1857, Signoret taxonomically differentiated *P. ficus* from *P. citri*, in light of their development on different host-plant species. In 1915 both were 1 reclassified as one species, but in 1956 they were again reclassified as two species [27], [28], [29]; Cox [6] separated them according to adult female morphology, and later their separation was further confirmed by the striking differences between the molecular structures of the female sex pheromones [25], [30], [31]. The *P. citri* pheromone consists of a single chemical component, i.e. (S+)-cis-(1R)-3-isopropenyl-2,2-imethylcyclobutanemethanol acetate [25], whereas *P. ficus* occurs in populations whose females release one pheromone compound, i.e. lavandulyl senecioate ( = LS) as well as other populations whose females release two pheromone compounds, i.e. LS and lavandulyl isovalerate ( = LI) [30], [31], [32]. *Planococcus ficus* and *P. citri* also differ in other properties (see Table 1), for example, development rate [24], [33], [34]. Recently molecular tools based on sequencing of the mitochondrial Cytochrome Oxidase I (COI) and the nuclear Internal Transcribed Spacer 2 (ITS2) genes [35], [36], [37] have been used to differentiate *P. citri* from *P. ficus*. Sequencing the ITS2 segments of suspected hybrid specimens is frequently used to demonstrate potential hybridization and gene flow between species [18], [38], [39], [40]. On the other hand, sequencing of maternally inherited COI segments is considered a better tool for phylogeny and taxonomy even though introgression of these segments from one species to another sometimes occurs [41], [42], [43], [44], [45].

**Table 1:** Summary of the main characteristics that distinguish between *P. citri* and *P. ficus*.

Character	*P. citri*	*P. ficus*	References
Origin	China or Africa*	Mediterranean	[26]
Host range	70 botanical families (mainly on non-woody parts)	12 botanical families (mainly on woody parts)	[88], [26]
Female morphology	0–35 multilocular disc pores on head; Tubular ducts: 0–30 on thorax; 0–35 on head; Cerarian setae on head and thorax – conical	0–4 multilocular disc pores on head; 0–4 tubular ducts on thorax; Cerarian setae on head and thorax – flagellate	[27], [28]
Male morphology	1–2 medial pronotal pores	3–6 medial pronotal pores	[89]
Development time	4–5 annual generations on citrus; Egg to oviposition on potato sprouts: 29 and; 37 days at 25.5 and 24.5°C, respectively.	3 annual generations on grapevines. Egg to adult female on grapevines: 25 days at 25°C.	[24], [26], [90]
Sex pheromones	cis-(1R)-3-isopropenyl-2,2-methylcyclobutanemethanol acetate	(S)-Lavandulyl senecioate ( = LS); (S)-Lavandulyl isovalerate ( = LI)	[25], [30], [31]
COI DNA sequencing	92 to 93% identity	92 to 93% identity	[36], [91], [37]
ITS2 DNA sequencing	90% identity	90% identity	[37]
Disease transmission	Grapevine Leaf Roll Virus (in the lab); Banana streak virus (BSV); Piper yellow mottle virus	Grapevine Leaf Roll Virus; Banana streak virus (BSV)	[92], [93], [94], [95]
Major crops affected	Subtropical orchard and greenhouse crops	Vineyards and fig, (Ficus carica) orchards	[96]

* Speculative data – based mainly on the suggested origin of the principal parasitic wasp of *P. citri*, especially *Leptomastix dactylopii* (60 Anga and Noyes 1999).

The natural occurrence of inter-species hybrids in insects is well documented [14], [15], [17], [41], [46], [47]. Among the scale insects (Coccoidea), laboratory-induced inter-species hybrids have been observed in the mealybug family (Pseudococcidae) [19], [20], [21], [22], [48].

However, evidence is yet lacking for natural gene flow between feral populations of sympatric mealybug species, or in other scale insect families. Hybrids of *P. citri* and *P. ficus* were documented in laboratory experiments by Tranfaglia and Tremblay [21], who found that the hybrid females displayed intermediate morphological features. By using crude solutions of pheromone effluvia of the two species, Rotundo and Tremblay [20] found that the laboratory hybrid males of each species displayed reciprocal attraction to the other congener pheromone in addition to that of conspecific females.

The present study aimed at revealing possible gene flow between feral populations of *P. citri* and *P. ficus*. We used sex pheromone male attraction and sequencing and GenBank references comparison of ITS2 and COI segments to show gene flow between those species. So far there is no evidence of field occurrence of natural hybrids of *P. citri* and *P. ficus* that could indicate occurrence of gene transfer between these species. However, in a controlled environment in the laboratory, these species are easily hybridized (20, Kol-Maimon et al., unpublished data). In many areas feral populations of these species share habitats, therefore cross-mating should be expected, although mealybug F1 hybrids may suffer marked mortality [48]. Rotundo and Tremblay 1982 [20] and Kol-Maimon et al. (unpublished data) documented high mortality among hybrid male crawlers of *P. citri* and *P. ficus*. Furthermore, no information about fertility of these F1 hybrids is available.

The objectives of this study were addressed by examining populations of *P. citri* and *P. ficus* from different locations and different host plants. Each population was characterized by its male pherotype behavior [32] and its genetic identity, e.g., COI, and by using molecular markers for detecting potential female hybrids that might account for pheromone cross-attraction of their male offspring. Furthermore, the ITS2 segments of suspected crossbred specimens were sequenced. This approach is frequently used to demonstrate potential hybridization and gene flow between 22 species, among both animals and plants [47], [38], [39], [40], [49].

## Materials and Methods

### Mealybug rearing

Unless otherwise specified, the mealybug populations were reared on potato sprouts washed with 95% ethanol in darkness, at 25o29 C and 50% relative humidity. Gravid females were transferred to clean potato sprouts, , and placed on tissue paper in sealed plastic containers measuring 15×10 cm in diameter and height, respectively, and covered with thin, polyethylene sheets which allow ventilation but prevent movement of crawlers between samples. Every 3 days the seal was removed for 5–10 min for ventilation, and the tissue papers were replaced to prevent development 1 of excess humidity and mold.

### Male pherotype characterization

The male pherotypes were characterized according to their specific responses to *P. ficus* and *P. citri* pheromones. Individual males were exposed to these compounds in no-choice tests, in which the compounds were presented in a random succession of three arenas [10]. The pheromone solutions for the bioassay were prepared by dissolving the appropriate pheromone component in n-hexane (1 ng/µl). The males were exposed in 10–cm-diameter glass Petri dish arenas, each containing a 5-mm-diameter filter paper disk (double-layer Whatman No 1) impregnated with 6 ng of the tested pheromone, and two untreated paper disks that served as controls. Pherotype identity was assigned to every male as follows: F – attracted to one or both *P. ficus* pheromones [31], [32]; C – attracted only to *P. citri* pheromone; FC – attracted to pheromones of both species; N – no attraction to any of the tested pheromones. Among those labeled as F and FC, we distinguish between males that responded to lavandulyl senecioate (LS) and those that responded to lavandulyl isovalerate (LI) or both. Further pherotypes that were identified: S20 attracted only to LS; I- attracted only to LI; SI- attracted to both LI and LS; CS21 attracted to both *P. citri* pheromone and LS; CI- attracted to both *P. citri* pheromone and LI; CSI- attracted to *P. citri* pheromone, LS and LI.

### Distribution of male pherotypes among the studied populations

Young and gravid mealybug females were collected in several different locations from different host plants (Table 2) and were placed on sprouted potatoes for further rearing. The male pherotype of the first laboratory-reared population was characterized as described above.

**Table 2:** List of the studied populations with respect to their origins and host plants.

Species	No.	Host plant, scientific and common name	Country of origin and location	Coordinates and elevation (m)
*P. citri*	1	Annona squamosa	Sicily, Catania	37°28′N, 14°75′E, 62
	2	Artemisia dracunculus, Taragon,*	Israel, Tomer	32°01′N, 35°44′E, 252
	3	Citrus grandis, Pomello,	Israel, Gilboa	32°31′N, 35°06′E, 102
	4	Citrus limon, Lemon	Spain, Mursia	37°59′N, 1°48′E, 39
	5	Citrus paradisi, Grapefruit	USA, UCR campus California	33°58′N, 117°19′W, 351
	6	Citrus reticulata, Clementine*	Israel, Bnei Zion	32°13′N, 34°60′E, 39
	7	Citrus sinensis, Orange	Portugal, Silves	37°12′N, 8°11′W, 48
	8	Diospyros kaki, Persimmon	Israel, Ein Shemer	32°28′N, 35°14′E, 49
	9	Mentha spicata, Mint*	Israel, Tomer	32°01′N, 35°44′E, 252
	10	Musa acuminata, Banana	Israel, Ein Ayala	32°38′N, 34°20′E, 18
	11	Punica granatum, Pomegranate	Israel, Iron	32°29′N, 34°71′E, 48
	12	Rosmarinus officinalis, Rosemary*	Israel, Na'ama	33°54′N, 35°96′E, 230
	13	Solanum tuberosum, Potato	Turkey, Antakya	Lab rearing, original host unknown
	14	Solenostemon sp., Coleus	USA, Logan Utah	41°44′N 111°48′W, ?
	15	Theobroma cacao, Cacao	USA, Miami Florida	(Subtropical Horticultural Research Station)
*P. ficus*	16	Ficus carica, Fig tree	Israel, Hula Valley	33°07′N, 35°18′E, 98
	17	Ficus carica, Fig tree	Israel, Elkosh	32°02′N, 35°43′E, 643
	18	Punica granatum, Pomegranate	Israel, Yotveta	26°53′N, 35°23′E, 77
	19	Punica granatum, Pomegranate	Turkey, Delibekirli	36°32′N; 36°19′E, 578
	20	Vitis vinifera, Grapevine (originally)	California, Parlier	Insectary rearing on butternut squash
	21	Same as 20	Israel, Sde Moshe	31°36′N, 34°19′E, 149
	22	Same as 20.	Israel, Meron	32°59′N, 35°62′E, 679
	23	Same as 20	Israel, Ma'a nit	32°26′N, 35°74′E, 46
	24	Same as 20	Israel, Odem	33°10′N, 35°64′E, 1076
	25	Same as 20	Sicily, Catania	37°28′N, 14°75′E, 62
	26	Same as 20.	Portugal, Tavira	37°6′N, 7°39′W, 25
	27	Same as 20	Spain, Mursia	37°59′N, 1°92′E, 38

* Commercial greenhouse.

### DNA extraction

Females were individually homogenized in 100 µl of CTAB (cetyl-trimethyl-ammonium bromide) buffer (1 M Tris; 5 M NaCl; 0.5 M EDTA; CTAB 2%; β-mercaptoethanol). The extract was incubated at 65°C for 2 h, after which DNA was extracted with chloroform (100%), and precipitated overnight with 8% ammonium acetate and 60% isopropanol. After maximum speed centrifugation for 15 min, the DNA pellet was washed with 70% ethanol, air-dried and resuspended in double-distilled water.

### Polymerase Chain Reaction (PCR) on COI and ITS2 gene segments

DNA at 100–500 ng/µl from each extraction was used for PCR. The PCR reaction (total volume of 50 µl) included: 42 µl of double-distilled water, 5 µl of Taq polymerase buffer, 0.6 µl of 20 pmole (0.24 pmole final concentration) primer 1 (COI-TL2-N-3014 or ITS2-M-R-454847), 0.6 µl of 20 pmole (0.24 pmole final concentration) primer 2 (COI- CJ-J-2183 or ITS2-M-F-454845), 0.4 µl of 25 mM dNTPs (0.2 mM final concentration), and 0.4 µl of 5 U/µl Taq DNA polymerase (0.08 U/µl final concentration). The cycling conditions were as follows: 95°C for 3 min, 35 cycles of 95°C for 1 min, 55°C for 1 min and 72°C for 1 min; and a final elongation cycle at 72°C for 10 min. The primers were: ITS2-M-F-454845: 5′ CTC GTG ACC AAA GAG TCC TG 3′. ITS2-M-R- 454847 5′ TGC TTA AGT TCA GCG GGT AG 3′. COI- CJ-J-2183: 5′ CAA CAT TTA TTT TGA TTT TTT 3′. COI- TL2-N-3014: 5′ TCC ATT GCA CTA ATC TGC CAT 3′. The primer sequences and PCR procedure were according to Malausa et al. 2010 [37]. Total size of fragments amplified was ∼800 bp for COI and ITS2.

### Cloning and sequencing of PCR products

The number of single nucleotide polymorphisms (SNPs) between *P.citri* and *P.ficus* on the ITS2 segment that was amplified in this study using BioEdit and ClustalW software is 58. ITS2 segment that was used to identify *P.citri* from *P.ficus* was ∼800 bp in length. 58 SNPs out of 800 bp is approximately 8%, which means 92% identity between those species of the ITS2 segment was used in this study. Comparison of ITS2 and COI sequences between different *P. citri* specimens and between different *P. ficus* specimens from GenBank data base show 98% identity and more within each species.

**Figure 1:**
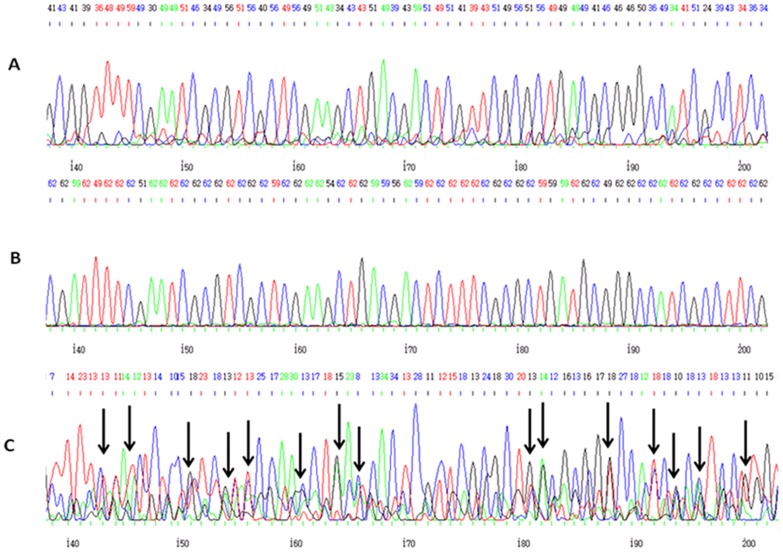
ITS2 sequences amplified from three types of females. A- Individuals with more than 98% identity with *P. citri* GenBank references, considered as *P. citri*. B- Individuals with more than 98% identity with *P. ficus* GenBank references, considered as *P. ficus*. H- Individuals with less than 92% identity with *P. citri* and *P. ficus* GenBank references, confirmed as hybrids of the two species by cloning sequencing. Black arrows mark double-peak signals indicating the existence of heterozygosity in this region. ITS2 GenBank references: *P. ficus*: GU134677, JQ085574, HQ852471; *P. citri*: JF714195. COI GenBank references: *P. ficus*: JN120845, EU250573, DQ238220; *P. citri*: AB439517, AF483204.

ITS2 sequences of *P. ficus* COI-verified females that showed more than 98% identity with the *P. ficus* GenBank reference [16], [29], [30] were considered as type A; ITS2 sequences of *P. citri* COI-verified females that showed more than 98% identity with the *P. citri* GenBank reference [31] were considered as type B; and those with less than 92% identity to *P. citri* or *P. ficus* GenBank references were considered as type H (Fig. 1). H type specimens were not detected among COI-verified *P. ficus* females.

To determine whether an ITS2 PCR product with double pick signals lengthwise (type H figure 1) was a hybrid DNA of the two species, the products of 7 out of 15 H type specimens were cloned into pGEM-T Easy vector (Promega, Madison, USA) according to the manufacturer's instructions. A total of 7 type H PCR products were cloned (according to table 3) and 5 clones were sequenced for each product. Following transformations and colony picking, individual clones were subjected to sequencing by Macrogen Inc., Seoul, Korea). For direct sequencing of PCR products, the products were run on 1% agarose gel and the expected band (size ∼800 bp) was excised from the gel and cleaned with the Qiaquick Gel Extraction Kit (Qiagen, Hilden, Germany). The cleaned DNA samples were sequenced by Macrogen, and the sequences were compared with the non-redundant (nr) nucleotide database in GenBank to verify the identity of the sequenced DNA.

**Table 3:** ITS2 sequencing identity according to GenBank references of females from various locations.

COI identity	Population (and number of tested individuals)	Type A	Type B	Type H	
	Outdoor habitat			Number	After cloning and sequencing
CM	2)* Tarragon (6)**	4	0	2	CM+VM
	3) Pomelo (6)	6	0	0	-
	4) Lemon Spain (9)	8	0	1	***
	5) Grapefruit USA (8)	7	0	1	***
	6) Clementine (6)	4	0	2	CM+VM
	7) Orange, Portugal (6)	6	0	0	-
	8) Persimmon (3)	2	0	1	CM+VM
	9) Mint (6)	5	0	1	CM+VM
	10) Banana (5)	4	0	1	CM+VM
	11) Pomegranate (9)	6	0	3	***
	12) Rosemary (4)	5	0	0	-
	14) Coleus, USA (9)	7	0	2	***
	15) Cacao, USA (10)	8	0	2	***
VM	16) Fig tree (10)	0	10	0	-
	17) Fig tree (10)	0	10	0	-
	18) Pomegranate (12)	0	12	0	-
	20) Grape vine, California (9)	0	9	0	-
	12) Grape vine (2)	0	2	0	-
	22) Grape vine (3)	0	3	0	-
	23) Grape vine (10)	0	10	0	-
	24) Grape vine (7)	0	7	0	-
	25) Grape vine, Sicily (7)	0	7	0	-
	26) Grape vine, Portugal (6)	0	6	0	-
	27) Grape vine, Spain (7)	0	7	0	-

The females were divided in to three groups: A- Individuals with more than 98% identity with *P. citri*, considered as *P. citri*. B- Individuals with more than 98% identity with *P. ficus*, considered as *P. ficus*. H- Individuals with less than 92% identity with *P. citri* and *P. ficus*, confirmed after cloning and sequencing as hybrids of the two species (CM refers to *P. citri* and VM to *P. ficus*). ITS2 GenBank references: *P. ficus*: GU134677, JQ085574, HQ852471, *P. citri*: JF714195. The species of the populations determined by COI sequencing and comparison to GenBank references (*P. ficus*: JN120845, EU250573, DQ238220 and *P. citri*: AB439517, AF483204).

* Numbering according to Table 2.

** Number of examined specimens.

*** Cloning could not obtained.

### Statistical analysis

Pearson contingency analysis was conducted for comparison of pherotype distribution between and within populations, by using JMP software version 7 (SAS Institute Inc., Cary, NC, USA). One-way MANOVA was conducted with the same software, for comparison among females with differing ITS2 sequence identities that were sampled from different populations.

## Results

### Pherotype distribution among mealybug populations from different habitats

**Figure 2:**
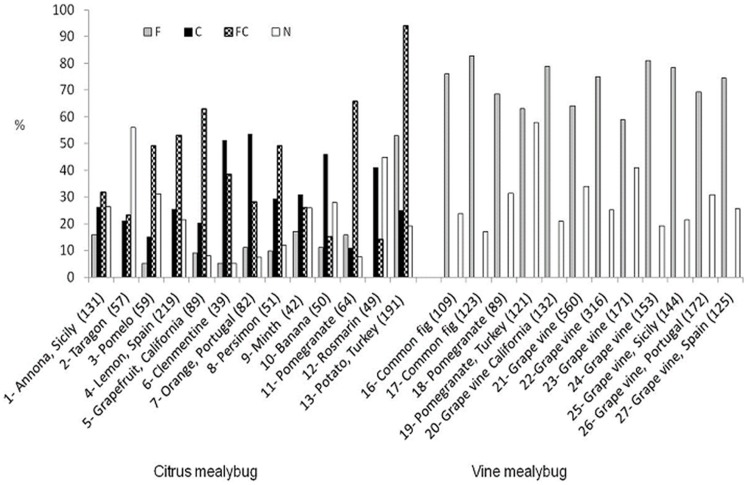
Pherotype frequency distribution among males whose mothers (gravid females) were sampled from various locations (see **Table 1**). The males were divided into four groups: (1) F- males – attracted to one or both *P. ficus* pheromones; (2) C- males – attracted only to *P. citri* pheromone; (3) FC- males – attracted to *P. citri* pheromone and also to one or both of *P. ficus* pheromones; (4) N- males – not attracted to any of the tested pheromones. Unless otherwise specified, the sampled hosts originated from Israel. The numbers preceding the host names are the serial numbers shown in Table 2; the numbers in parentheses are the numbers of tested males per tested population. The species of the populations were determined by COI sequencing and comparison with gene bank references (*P. ficus*: JN120845, EU250573, DQ238220; *P. citri*: AB439517, AF483204).

Male pherotypes and their distributions among the sampled populations from diverse locations and various host plants, arranged according to mealybug species are displayed in Fig. 2. All examined *P. citri* populations included male pherotypes that were attracted to either *P. citri* or *P. ficus* pheromones, or both. Males attracted solely to the sex pheromone of *P. ficus* were detected in 20 out of the 23 tested populations of *P. citri*. In all tested populations of each species considerable numbers of males were indifferent to all three tested pheromones, i.e., they were N pherotype. Among *P. citri* populations, an average of 29.8% of the males (range, 11–54%) were C pherotype, i.e., attracted to *P. citri* pheromone only; 39.4% of the males (range, 14–66%) were FC, i.e., attracted to both *P. citri* and *P. ficus* pheromones; 9.3% of the males (range, 0–13%) were F pherotype, i.e., attracted only to *P. ficus* pheromones; and the remaining 21.4% (range, 5–56%) were N pherotype, i.e., showing no attraction to either of the tested pheromones. All 11 examined *P. ficus* populations consisted of male pherotypes that were attracted to *P. ficus* pheromones (LS, LI or both). On average, 70.4% (range, 52–83%) of males of the *P. ficus* populations were F pherotype and the remaining 29.6% (range, 17–48%) were N pherotype. Pearson contingency analysis showed that pherotype distributions differed significantly between the *P. citri* populations (*P*<0.0001; χ^2^ = 310.7 between hosts; *P*<0.0001; χ^2^ = 291.8 between origins) and within *P. ficus* populations (*P*<0.001; χ^2^ = 10.8 between hosts; *P*<0.0001; χ^2^ = 49.7 between origins), but a significantly larger distance was found between species (*P*<0.0001; χ^2^ = 1793) than within species. Pearson contingency analysis further showed a larger variance or heterogeneity within the *P. citri* group than within the *P. ficus* group, probably because of higher pherotype variability among males of the former group.

Among male *P. citri* populations from the East Mediterranean region, pherotypes that were attracted to *P. citri* pheromone and to LS (i.e., C, S and CS pherotypes) comprised 67 and 71% of those from Israel and Turkey, respectively; those that were attracted to LI pheromone, alone or in combination with *P. citri* pheromone, and to *P. ficus* pheromone (i.e., I, SI, CI and CSI) comprised 25 and 19% of those from Israel and Turkey, respectively; and those that were characterized as not attracted to any pheromone (i.e., N) comprised 8 and 10% of the tested males, from Israel and Turkey, respectively. *Planococcus ficus* males of the East Mediterranean populations consisted of pherotypes that were attracted to LS pheromone (S) – 33 and 19% from Israel and Turkey respectively; those that were attracted to LI, alone or in combination with LS (I, SI) – 41 and 33% from Israel and Turkey, respectively. The N males consisted of 26 and 48% of the sampled populations from Israel and Turkey respectively.

*Planococcus citri* males from the American, west- and mid-Mediterranean populations consisted of pherotypes that were attracted to *P. citri* pheromone and others that were attracted to LS pheromones (C, S, CS); they formed 92, 84, 77 and 67% of the male populations from California, Portugal, Spain and Sicily, respectively; and unnattracted males (N) males formed 8, 16, 23 and 33%, respectively, of the populations from these areas. Among *Planococcus ficus* males of the Californian, mid- and west-Mediterranean populations, pherotypes that were attracted to LS pheromone (S) formed 79, 70, 73 and 79% of the populations from California, Portugal, Spain and Sicily, respectively; and N males formed 21, 30, 27 and 21%, respectively, of these populations. No male attraction to LI pheromone was detected among Californian, west- and mid-Mediterranean populations of either *P. citri* or *P. ficus*, whereas males that were attracted to LI were found among east-Mediterranean populations of both *P. citri* and *P. ficus*.

For statistical analysis all males attracted to LI, alone or in combination with I, SI, CSI or CI, were considered as one pherotype group, designated as the I-group; males that were not attracted to LI (i.e., groups S, C, CS, N) were considered as a second pherotype group, designated the Non-I group. Pearson contingency analysis showed significant differences between *P. citri* and *P. ficus* (*P*<0.0001; χ^2^ = 57.7), in the distributions between the I-group and the Non-I group. A significant and larger distance between I pherotypes and Non-I pherotypes was observed among east-Mediterranean populations than among west- and mid-Mediterranean populations (*P*<0.0001; χ^2^ = 337.3). No significant differences were found within the group with LI attraction, in east-Mediterranean populations (*P* = 0.15; χ^2^ = 2.1). No attraction to LI was found among American, west- and mid-Mediterranean populations. The distribution of male attraction to LI was influenced by the species but more strongly by the area of origin.

### Genetic identity of the tested mealybug populations

Out of 13 tested *P. citri* populations 11 contained females that showed less than 92% identity to *P. citri* and *P. ficus* GenBank references (type H in Fig. 1) but such females were not detected among *P. ficus* populations (Table 3). In addition, the nucleotide chromatograms of all H females showed double-peak signals lengthwise in each sequence (as indicated by arrows in Fig. 1), indicating that the sequencing reactions suggested the presence of two PCR products. Following cloning and sequencing of the pair of ITS2 sequences of some of the type H females, comparison with GenBank references [37], [50], [51] revealed that one member of the ITS2 pair displayed ≥98% similarity to either *P. citri* or *P. ficus*, therefore the ITS2 sequences of H females are likely to represent hybridization between *P. citri* and *P. ficus*.

One-way MANOVA revealed a significant difference between *P. citri* and *P. ficus* populations in their ITS2 identities (types A, B, H as described in Fig. 1) (*P*<0.0001; *F*_1_ = 70.3439, *P*<0.0001; *F*_1_ = 90.2481, *P*<0.0001; *F*_1_ = 23.1465; for types A, B, and H respectively). These results reveal a clear relationship, in the studied mealybug species, between the type of species and the ITS2 identities of females. On the other hand, the one-way MANOVA suggested no significant differences of ITS2 identity between the studied populations from different origins (*P* = 0.7; *F*_4_ = 0.6, *P* = 0.08; *F*_4_ = 2.5, *P* = 0.3; *F*_4_ = 1.4; for types A, B, and H, respectively) or different host plants (*P* = 0.3; *F*_14_ = 1.4, *P* = 0.2; *F*_14_ = 1.8; for types A and H respectively). These findings imply that neither host plant nor sampling area factors were related to ITS2 identity of females in the tested populations.

## Discussion

The two mealybug species addressed in the present study – *P. ficus* and *P. citri* – belong to the ‘*citri*’ species group of the genus *Planococcus* [52] and they are closely genetically related [35], [37], [53]. The sex pheromones of three among the 12 members of the ‘*citri*’ group were previously identified as those of the species addressed in the present study – *ficus* and *citri* – and that of *Planococcus minor* [53]. The sex pheromone compounds of these three species are structurally different [25], [30], [31], [53], therefore, the cross attraction between *P. ficus* and *P. citri* was unexpected. Insect sex-pheromone signals are highly species-specific, as recorded in aphids [54], moths [55], [56] and in scale insects [23]. However, cross-attraction between congeners is a common, but not always clear, phenomenon; it may result from very similar or shared identical sex pheromone compounds [56], [57], [58], [59] which does not apply to the presently studied mealybug species. Cross-attraction between the closely related ermine moth species *Yponomeuta* spp. was reported by Hendrikse 1988 [59]; and later Löfstedt et al. 1991 [60] suggested that cross-attraction between allochronic species of ermine moths in the laboratory, and the formation of hybrids in the laboratory between species that are reproductively isolated by pheromone differences, is evidence for the role of pheromones as reproductive isolation mechanisms among these moth species. Little is known about the genetic mechanisms that underlie the evolution of new sex pheromones between related species and still less with regard to specific olfactory pheromone receptors. However, it is to be expected that the male preference for a sex pheromone should co-evolve in parallel with the female sex pheromones [61]. Pre-mating barriers, such as lack of pheromone attraction, may be the most important factors determining reproductive isolation in many taxa [62], but occurrence of hybrids may be a reason for cross-attraction. In the European corn borer moth, *Ostrinia nubilalis* (Crambidae) there are two pheromone-related strains – Z and E – which produce and respond to different E/Z-11-tetradecenyl acetate ratios [63]. In the laboratory E and Z borer strains can interbreed [64] but, in spite of the use of the same sex pheromone blend, cross-attraction between these moths was rare. However, hybrid males produced in the laboratory responded over a broad range of Z and E pheromone blends [65]. Rotundo and Tremblay 1982 [20] were the first to show that laboratory-produced hybrid males of *P. ficus* and *P. citri* displayed an attraction to their maternal sex pheromone. However, the small number of hybrid males produced in that study probably prevented the occurrence of males attracted to their paternal-line sex pheromone.

In the present study we found that certain proportions (23–82%) of male pherotypes produced by any of the tested *P. citri* populations were attracted to the *P. ficus* pheromone. However, no male pherotypes attracted to *P. citri* pheromone were detected in any of the tested populations of *P. ficus*. This phenotypic picture is meaningful because the tested populations were taken from different areas and different host plants. The genetic aspect of this phenomenon is indirectly supported by cloning and sequencing of ITS2 DNA fragments, which revealed the existence of hybrid females in most of the sampled *P. citri* populations. It is suggested that in three out of 13 tested *P. citri* populations, these hybrids were not detected because of the small sample size and a minority of them in those cases (males in those populations were attracted to *P. ficus* pheromones); similarly, hybrid females were not detected among the tested *P. ficus* populations. The ITS2 gene sequence is highly preserved within species, because of its significant function among all organisms, as a ribosomal gene [66]. Therefore, the occurrence of ITS2 sequence polymorphism among populations. Because of its significant function among all organisms, the supported by cloning and sequencing, is an indication of gene flow, probably through hybridization between species [67], [44], [68].

One explanation for the occurrence of pherotypes of *P. citri* males that are attracted to the pheromone of *P. ficus* may be the occurrence of gene flow from *P. ficus* to *P. citri*. These species share the same habitate landscape in many areas. For example, in various Mediterranean-climatic areas, the widespread host plants of the presently studied mealybug species are citrus groves that support large population of *P. citri*, and that grow adjacent to vineyards, fig (*Ficus carica*) and pomegranate trees that harbor the vine mealybug. The observation of populations of *P. citri* and *P. ficus* hybrids on banana and plantain in Uganda, as suggested by Watson and Kubiriba 2005 [69], was based on female morphology. However, intermediate female morphology could be seen in populations growing under extreme temperatures [28]. Thus, so far no conclusive evidence was found in the field for the occurrence of natural hybrids of *P. citri* and *P. ficus*, or of any other scale insect species, which could show contemporary gene flow. The possible existence of inter-species hybrids is also often ruled out because of low or absence of fitness, as expressed in sterility, host incompatibility, and other factors [13], [70]. Production of hybrids in an artificial environment may be possible because the usual ecological isolation can be breached under laboratory conditions by interbreeding [2], [4].

Laboratory crosses between *Pseudococcus* spp. and *Phenococcus* spp. and *P. citri* produced no viable adult offspring [48]. However, *P. citri* and *P. ficus* are easily hybridized in the laboratory, and the hybrids are fertile to some extent [20], therefore, the possible occurrence of natural populations including hybrids should be taken into account. It is interesting to note that laboratory-generated hybrids that resulted from crossing *P. ficus* females with *P. citri* males resulted in high mortality, as compared with lower mortality of the reciprocal crossing (Kol-Maimon et al., unpublished data). In general, interspecific hybrids are less fit than thoroughbreds because of the large number of changes in their genomes [71]. Although there is no information on the occurrence of hybrid scale insects in the wild, we may assume that survival of hybrids on wild plants would be even less probable than their survival on potato sprouts in the laboratory. Offspring produced as a result of cross-mating between *P. ficus* females and *P. citri* males (unlike the reciprocal cross-mating) hardly ever survive in the laboratory, therefore, *P. ficus* males displaying characteristics of *P. citri* males are unlikely to complete their development on plants in the field. This directional gene flow, from *P. ficus* to *P. citri*, but not vice versa, is a meaningful topic for future research on reproduction and genetic systems in mealybugs.

The presence of similar traits and gene sequences among two different species is not necessarily evidence for contemporary gene flow between species [72], [73], [74]. The question is whether the occurrence of a ‘hybrid *P. citri*’ population, as shown in our present study, is a modern event brought about by anthropogenic activity, or whether, in fact, *P. ficus* resulted from sympatric speciation. Both mealybug species almost certainly share a common ancestor [52]. The phylogenic relationships among the 12 species of the ‘*citri*’ group [75] are not clear. In light of information on the ‘*citri*’ members accumulated by Cox 1989 [75], it seems that the origin of the group is in Africa. According to Rung et al. [35], *P. minor* is genetically closer to *P. citri* than is *P. ficus*. The distribution of *P. minor* was discussed by Rung et al. [35], who emphasized the fact that this species is absent from the African mainland; they suggested that the origin of *P. minor* is in the eastern Palaearctic, which suggests that the speciation of *P. citri* and *P. minor* may have happened in Asia. The area of origin of *P. citri* is speculative, and ideas about it were derived from the areas from which the four principle parasitoids of the mealybug were first collected. *Leptomastix dactylopii* was believed to be from South America [76]. However, it was suggested that *L. dactylopii* spread to South America during the era of the slave trade, because it belongs to the fauna of the African *Leptomastix* spp. [77]. The origin of another major parasitoid, *Anagyrus pseudococci* (a complex of two species or sub-species) [78] is the Mediterranean Basin [79]. The latter area is also the suggested origin of *Leptomastidea abnormis* [80]. *Coccidoxenoides perminutus* is believed to originate from southern China [81] or Australia [82]. All these four parasitoid species are known from populations of *P. ficus* in the Mediterranean area; *A. pseudococci* is attracted to the female sex pheromone of *P. ficus* but not to that of *P. citri* [83], [84]. This information suggests that *P. citri*, unlike *P. ficus*, was not originally associated with *L. dactylopii* or *A. pseudococci*. The area of origin of *P. ficus* is not discussed in the literature, although many authors claim the Mediterranean basin to be its area of origin. Miller et al. 2005 [84] suggested that it belonged to the Palearctic region. The natural distribution of *P. ficus* was accepted to be the Mediterranean Basin, but it easily could be the larger area that includes East Africa. If the natural area of *P. citri*, similarly to that of *P. minor*, is the East Palearctic, then it is more likely that the occurrence of *P. ficus* characteristics among *P. citri* populations is a result of a modern process related to transfer of these mealybugs to these same areas on cultivated plant species.

However, the question of whether the separation between *P. citri* and *P. ficus* is a result of sympatric or allopatric speciation is still open; shared traits among sympatric and allopatric populations of the same species are evidence for ancient rather than contemporary gene flow [73]. Other studies suggest that introgression could be a significant source of genetic variation in hybridizing species groups [85]. Qualitative manifestations of introgression may appear to be most impressive when hybridizing species meet in zones of secondary contact, following a period of divergence and lineage sorting in allopatry [86], and this may apply to the two presently studied mealybug species. The widespread occurrence of male pherotypes attracted to the *P. ficus* pheromone lavandulyl senecioate, i.e., LS males, in all *P. citri* populations (Table 4) might suggest that development of this pherotypic male structure could be a modern event. The occurrence of *P. citri* males attracted to lavandulyl isovalerate, i.e., LI males, among the East Mediterranean population of *P. citri* (Table 4) suggests a recent gene flow from *P. ficus* to *P. citri* in this particular area. Lavandulyl isovalerate is a pheromone compound produced by *P. ficus* populations, and *P. ficus* males attracted to it were found only in East Mediterranean populations, as in the course of the present study as well as in previous research [31], [32], [87].

**Table 4:** Pherotype frequency distribution (mean %) among males.

Origin	Species	Pherotype (mean %)							
		I	SI	CSI	CI	C	CS	S	N
East Mediterranean countries (Israel and Turkey)	*P. citri*	1	4	5	2	30	42	7	9
	*P. ficus*	11	24	0	0	0	0	30	35
Mid-Mediterranean country (Sicily)	*P. citri*	0	0	0	0	38	14	15	33
	*P. ficus*	0	0	0	0	0	0	79	21
West Mediterranean countries (Portugal and Spain)	*P. citri*	0	0	0	0	39	38	39	15
	*P. ficus*	0	0	0	0	0	0	72	28
North America (California)	*P. citri*	0	0	0	0	20	63	9	8
	*P. ficus*	0	0	0	0	0	0	79	21

Males whose mothers (gravid females) were sampled from Mediterranean countries, with emphasis on attraction to *P. ficus* pheromone Lavandulyl isovalerate (LI). The males were divided into 8 groups according to their pherotypic characteristics. Letters represent: I – attraction to LI *P. ficus* pheromone; S – Attraction to LS *P. ficus* pheromone; C – attraction to *P. citri* pheromone; N – no attraction to either of the tested pheromones. Combinations of two or three letters per pherotype indicate attraction to two or three pheromones.

Phylogenetic study of the populations of *P. ficus* and *P. citri* may shed more light on the relationship between these populations (Fig. S1). The close genetic relationship between *P. citri* and *P. minor* led to the speculation that the existence of male pherotypes of *P. citri* that respond to the *P. minor* female sex pheromone cannot be ruled out. In both *P. citri* and *P. ficus* significant percentages of males did not respond to the pheromones produced by either of these species. The occurrence of such males was already addressed by Kol-Maimon et al. 2010 [32] with regard to *P. ficus*, and was demonstrated in the present study for both species in all tested populations. The question of whether these male pherotypes might respond to the pheromones of other close congeners, such as *P. minor* or *P. halli*, is a legitimate. Finally, we suggest that the pherotype issue might serve as an important tool for revealing genetic associations and past interaction(s) between scale insect congeners.

## Supporting Information

Figure S1 **Phylogenetic tree based on representative ITS2 sequences of the populations from** **table 3** **in the manuscript.** Host, country of origin (serial number from table 2 - GenBank accession number) Clone = After cloning sequence according to table 3, PC or PF = *Planococcus citri* or *Planococcus ficus* based on GenBank alignment. Branches colors: Red- PC, Blue- PF, Green- Clone.
(PDF)
