# Supplementary figures and images for: Evidence for Gene Flow between Two Sympatric Mealybug Species (Insecta; Coccoidea; Pseudococcidae)
Source: PLoS One. 2014 Feb 11;9(2):e88433. doi: 10.1371/journal.pone.0088433 (PMC3921159; doi:10.1371/journal.pone.0088433)

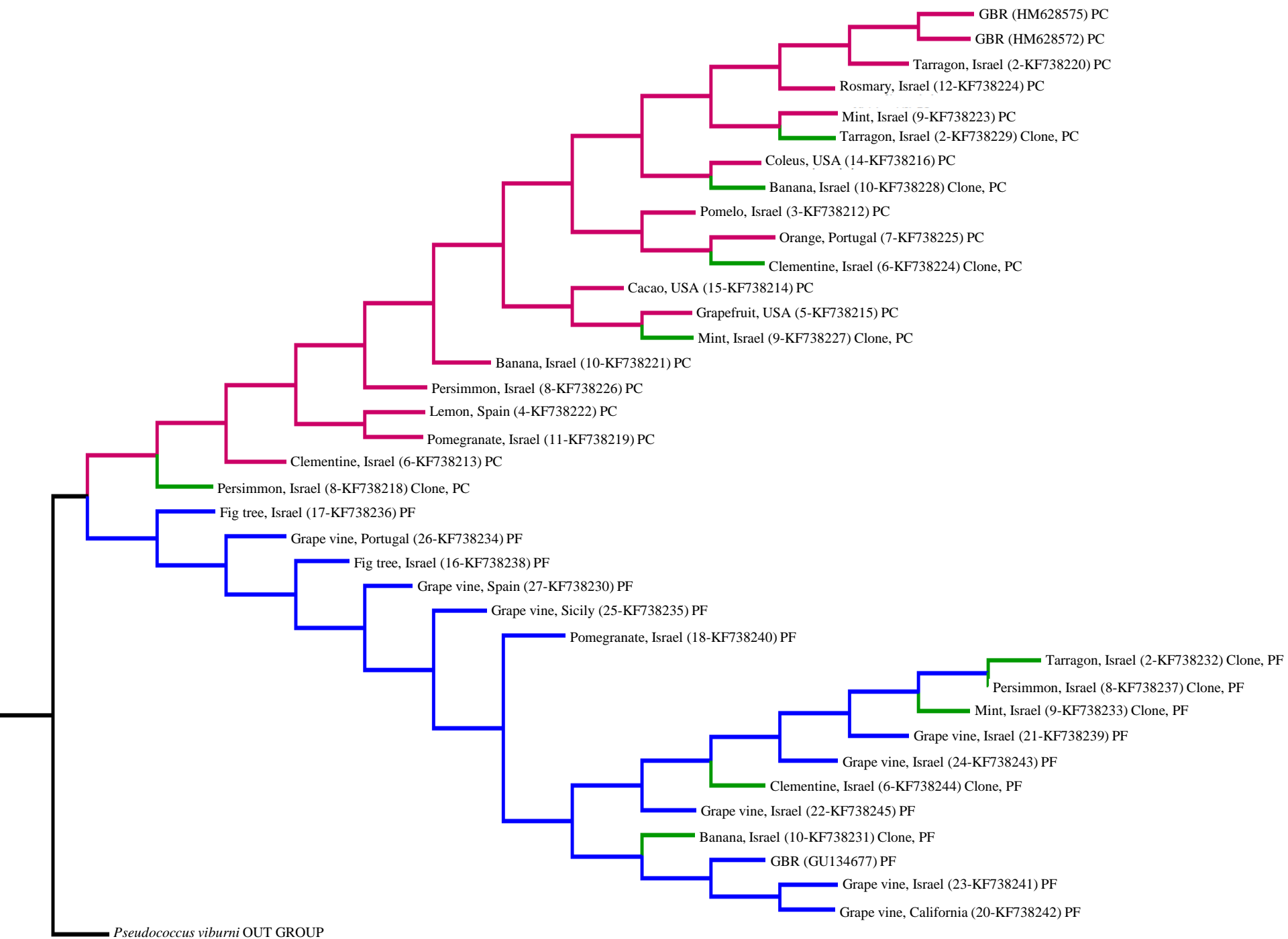

Supplement: Figure S1 — Phylogenetic tree based on representative ITS2 sequences of the populations from table 3 in the manuscript. Host, country of origin (serial number from table 2 - GenBank accession number) Clone = After cloning sequence according to table 3, PC or PF = Planococcus citri or Planococcus ficus based on GenBank alignment. Branches colors: Red- PC, Blue- PF, Green- Clone. (PDF) [file pone.0088433.s001.pdf]
